# Student selection cannot resolve the lack of general practitioners and country doctors

**DOI:** 10.3205/zma001093

**Published:** 2017-05-15

**Authors:** Johanna Hissbach, Stefan Zimmermann, Wolfgang Hampe

**Affiliations:** 1 University Medical Center Hamburg-Eppendorf, Department of Biochemistry and Molecular Cell Biology, Hamburg, Germany

## Letter to the Editor

In their article, Kesternich et al. [1] address the important topic of the shortage of general practitioners (GPs) and country doctors, which is considered in the “Masterplan Medizinstudium 2020” (master plan medical studies 2020) in the form of an admission quota for prospective country doctors. The study presents the results of a survey among medical students from Munich (Germany) in the clinical section of their studies. In a multivariate model Kesternich et al. determine the influence of socio-demographic factors, typical parameters considered in student selection procedures, and risk aversion on the intention to become a country doctor or a GP, respectively. Only

- a higher self-reported risk aversion,
- a lower Abitur grade (German high school GPA),
- as well as the fact that at least one parent is a medical doctor

are significantly and positively correlated with one or both intentions. Not significantly correlated are, amongst others, previous practical experiences in the health sector, the expected income, the First National Boards Examination grade (“Physikumsnote”), or the waiting period prior to the beginning of the studies. Unfortunately, the article does not include descriptive statistics for the subgroups, such as the number of students with waiting period who consider to become a country doctor (we assume that this group, which is important in the model, comprises only a few students). The quality of the model fit in the form of pseudo-R^2^ or in that of a classification table is not provided. Thus, an evaluation of the presented results is difficult.

In the discussion, the authors present a number of suggestions for future student selection procedures, aiming at an increase of GPs in rural areas. To achieve that, it is suggested to decrease the influence of the Abitur grade by including additional criteria to assess the motivation for medical studies:

1. Voluntary social commitment or work experience in the health sector as stated in the curriculum vitae: In the multivariate model, previous practical experience in the health sector was not predictive of the intention to work as a GP or a country doctor, data concerning social commitment are not reported.
2. Estimating the willingness to work as a GP in a rural area by means of multiple mini interviews (MMI): Even though MMIs are correlated with the evaluation of undergraduate practical medical work, we consider this suggestion – based on both international [2] and our own eight year experience with the Hamburg MMI – barely feasible to predict an unfeigned motivation or later choice of specialization among the still very young applicants. In addition, MMIs are very expensive and, under the given circumstances, viable only for a very small share of the applicants.
3. Consideration of semesters not enrolled at university, e. g. by increasing the waiting period quota: In the study, waiting semesters are not significantly correlated with the intention to become a GP or a country doctor, respectively. The study does not mention that in many German Universities, about 40% of the students from the waiting period quota do not pass the First National Boards Examination (“Physikum”) [3]. Thus, even if a higher percentage of students from this group indeed intended to work in rural areas, the effect would be obliterated by the high number of dropouts.

We are particularly critical of the following suggestion: “In particular, grade requirements for admission could be lowered for candidates who […] have a family background of rural medicine.” [1] (p. 7). The results of the survey imply that children of medical doctors develop the intention to become country doctors more often, but we believe that preferring doctors’ children is neither in accordance with the German Constitution, nor desirable considering the low permeability of our educational system.

Politics are confronted with the difficult problem of finding a remedy for the shortage of country doctors and GPs. From our point of view, the authors’ proposals are neither supported by their empirical findings, nor would they lead to the desired result. An expansion of the waiting period quota would result in a decrease of graduates, further exacerbating the shortage of country doctors. A Cochrane report [4] as well as recommendations of the WHO [5] consider one’s own provenance from rural areas as the only validated factor in the selection of students which indeed indicates a later occupation as a country doctor. Unfortunately, the current study does not include the corresponding question concerning the students’ rural origin. Moreover, selection based on the place of origin would be constitutionally questionable. The effectiveness of a country doctor quota, as discussed in the master plan, has not been examined yet. As the decision for the area of specialty training is usually made during the course of studies, measures promoting general medicine in the curriculum, as encouraged in the master plan as well, are able to raise the number of GPs [4], [5].

In our opinion, it is a sensible suggestion by Kesternich et al. to include “the specialization choice of students as a criterion when evaluating entry requirements.” [1] (p. 6). For this, we need longitudinal studies covering the time from university application to the choice of specialty training and work placement.

## Competing interests

The authors declare that they have no competing interests.
